# Tackling “pillar 5” – exploring options to help families of children who are living with congenital adrenal hyperplasia (CAH) in Vietnam to achieve financial independence

**DOI:** 10.1186/1687-9856-2013-S1-P136

**Published:** 2013-10-03

**Authors:** KL Armstrong, D Vu Chi, Nguyen Thanh Liem, S Ditchfield, C Cole, J Willett

**Affiliations:** 1 CLAN – Caring & Living As Neighbours, Australia; 2 National Hospital of Pediatrics (NHP), Hanoi, Vietnam

## Background

Despite significant improvements in care and health system strengthening for children who are living with Congenital Adrenal Hyperplasia (CAH) in Vietnam in recent years, consultation with families has confirmed that financial burdens continue to present urgent and pressing challenges for many. As part of its strategic framework for action and “5 Pillar” approach to helping children who are living in resource poor countries enjoy the highest quality of life possible, CLAN (Caring & Living As Neighbours) is committed to helping families achieve financial independence, and overcome any financial barriers to providing their children with the best care they can.

## Aim

This project sought to understand the financial burdens affecting CAH families in Vietnam and potential opportunities for the development of income generating activities.

## Method

The project concentrated on the most vulnerable and impoverished families. With support from members and staff connected with the CAH Club of NHP, willing families in rural areas of North Vietnam were identified for participation in face-to-face interviews in their homes in the month of July 2012. Families’ financial situation and possible avenues to improve same were discussed.

## Results

Approximately 50% of all 650 CAH families attending NHP for care live in rural areas. Interviews were conducted with 22 families, with good saturation of responses emerging. The income of most families was under 4,000,000VND ($AUD200) per month, and most were unable to save money. Two key costs for families emerged: that of Florinef and the cost of travel to attend NHP. All mothers interviewed had a network of 2-3 other mothers from the CAH Club with whom they regularly communicated for advice and support. The CAH Club was considered a vital resource. A technique for identifying which families should be helped was developed, and was based on income level and individual plans to improve financial status.

## Conclusion

It was estimated 35% (around 120) of the rural CAH families from NHP are in need of urgent priority support. Costs within and beyond the control of families were identified for focus. CLAN will continue to collaborate with NHP to explore most appropriate next steps for action.

